# Dynamic acoustic focusing in photoacoustic transmitter

**DOI:** 10.1016/j.pacs.2020.100224

**Published:** 2020-11-28

**Authors:** Qi Li, Jiapu Li, Haobo Zhu, Yujie Chen, Benpeng Zhu, Hongbin Yu

**Affiliations:** a School of Optical and Electronic Technology, China Jiliang University, Hangzhou 310018, China; b School of Optical and Electronic Information, Huazhong University of Science and Technology, Wuhan 430074, China; c Wuhan National Laboratory for Optoelectronics, Huazhong University of Science and Technology, Wuhan 430074, China

**Keywords:** Dynamic acoustic focusing, Photoacoustic transmitter, Candle soot nanoparticles (CSNPs), Polydimethylsiloxane (PDMS), Pneumatic actuator

## Abstract

Photoacoustic transmitter represents a promising substitute for conventional piezoelectric counterparts. However, lack of easy and effective method for dynamically manipulating the focused acoustic field is a common and tricky problem faced by current photoacoustic technology. In this paper, a new strategy for constructing focus tunable photoacoustic transmitter is proposed. Different from existed prevailing device architecture, a sandwich like photoacoustic conversion layer is innovatively designed into a suspending elastic membrane with clamped boundary and it can be deformed using integrated pneumatic actuator. Owing to the membrane deflection property, concave spherical contours with variable radius of curvature can be obtained. Considering the shape determined sound emission characteristic, continuous tuning on the axial focusing length of the acoustic field has been successfully demonstrated in the photoacoustic transmitter for the first time. Besides, acoustic signal with significantly improved negative pressure has also been achieved especially at the focus, bringing additional advantage for applications.

## Highlights

• Sandwich-like free-standing composite photoacoustic layer is used.
• Photoacoustic layer can be deformed into controllable concave spherical contour using integrated pneumatic actuator.
• Dynamic acoustic focal length tuning along axial direction is experimentally demonstrated for the first time.

## 1 Introduction

Benefit from the non-ionized radiation and large penetration depth characteristics, ultrasound has been widely used especially in biomedical applications as medical imaging [[1], [2], [3]], drug delivery [4], cell manipulation [[5], [6], [7]], lithotripsy [8] and sonothrombolysis [9]. In the ultrasound society, the electro-acoustic transducers represent dominant technology for sound generation through utilizing mechanical vibration of solid structure. More recently, the photoacoustic(PA) strategy, which creates ultrasound using PA effect is emerging as an attractive alternative to traditional counterparts due to its outstanding superiority in providing ultrasound signal with high frequency, broad bandwidth and high pressure amplitude [[10], [11], [12], [13], [14]]. In real applications, the acoustic field is usually required to be made tightly focused so as to improve the lateral resolution and further increase the sound pressure level via reduced acoustic distribution region and concentrated acoustic energy, respectively [[15], [16], [17], [18], [19], [20]]. As for the PA technology, the most commonly adopted method is to directly coat a PA conversion layer onto a solid transparent substrate with concave surface contour (such as glass concave lens) [[21], [22], [23]]. Unfortunately, this approach has to be faced with two prominent problems during operation. On the one hand, from fabrication point of view, it is a rather complicated process to coat a uniform PA conversion layer onto a curved surface, which is critical for device performance, thus making the control of performance uniformity and fabrication repeatability difficult. On the other hand, due to the fixed surface contour, the resultant focused acoustic field will also be predefined and kept unchanged during operation. However, considering the fact that the interested regions or the targets under treated usually posses certain depth along the acoustic wave transmission path. As a result, additional manipulation procedure is needed to provide manual axial scanning of the acoustic focus so as to cover the whole region. For example, in the case of ultrasound imaging, clear image with the best resolution and signal to noise ratio can only be obtained at the focal plane. In order to capture the entire 3D image, the axial scan of the focal plane is essential and it has to be achieved through moving the ultrasound probe due to its fixed focal length and limited depth of focus. Obviously, it puts forward higher requirement for operators and largely lowers the operation efficiency as well as accuracy.

Various efforts have been made to try to solve these issues and exciting results have been reported. For example, Yu etal proposed a novel strategy through combining mature planar coating process with elastomeric molding to make concave PA conversion layer [24]. As a result, the fabrication of focused PA transmitter can be largely simplified. At the same time, Wang etal designed optical fiber array to generate PA wave [25,26]. Different from conventional treatment that relies on the surface contour of the PA conversion layer, through properly arranging the length of individual fibers, certain acoustic field distribution can be obtained with flat fiber ends. In comparison to the remarkable progresses in fabrication process simplification, the status for treating the second issue associated with fixed device performance is a bit intricate. Although several concepts have been proposed [[27], [28], [29]], they either face technical obstacle or require too complicated and expensive hardware configuration as well as software control to be practical in use. Till now, unlike the electro-acoustic counterpart using electrically controlled phased array technology, no effective technical means has been successfully developed in PA area to experimentally demonstrate continuous tuning on ultrasound focusing yet.

**Fig. 1:**
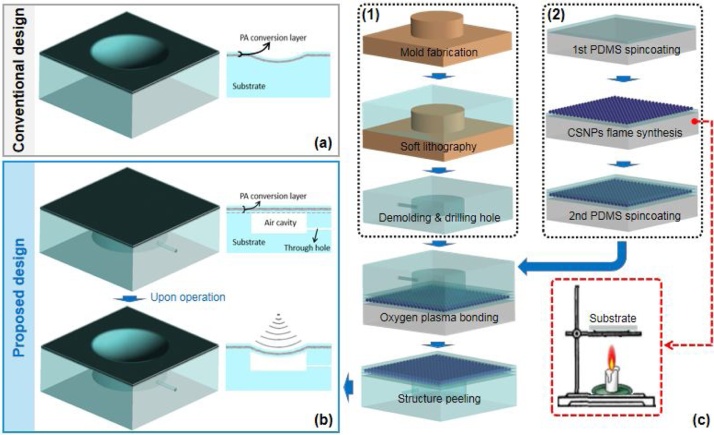
Details about the proposed strategy. (a) Conventional design for focused PA transmitter, in which the PA conversion layer is directly coated onto a solid substrate with concave surface. (b) Proposed design for focus tunable PA transmitter, in which a sandwich like PA conversion layer is made suspending over an air cavity to construct pneumatic actuator. Upon actuation, the PA layer will be forced to deform into concave spherical profile with variable radius of curvature. (c) Schematic of the fabrication process for the proposed device, it can be divided into two parts: (1) and (2) show the fabrication for the substrate and the PA conversion layer, respectively.

To address the technical unmet related to dynamic acoustic field focusing tuning, in this paper, a novel strategy for constructing focus tunable PA transmitter is presented. Distinct from direct coating of the PA conversion layer onto a solid substrate that being adopted in dominating device design (as shown in Fig. 1(a)), in current case, a sandwich-like free-standing PA conversion layer is developed and it is bonded to a PDMS substrate incorporating a cylinder cavity to form an integrated pneumatic actuator. Utilizing its mechanical deflection property, upon actuation, the originally flat PA layer suspending over the cavity will be deformed into a spherical like surface profile and its radius of curvature can be easily yet well controlled via actuation pressure (see Fig. 1(b)). When combining the shape determined sound emission distribution characteristic associated with PA technology, we experimentally demonstrate, to the best of our knowledge, the first dynamic acoustic focusing capability in PA transmitter.

## 2 Device fabrication

For proof-of-concept validation, composite incorporating candle soot nanoparticles(CSNPs) and polydimethylsiloxane(PDMS) is used to construct the PA conversion layer due to its high PA conversion efficiency and relatively facile fabrication process. Fig. 1(c) shows the fabrication process flow of the proposed PA transmitter. Firstly, anti-adhesive treatment for a clean glass slide is performed through silane coating for 3 h within a vacuum desiccator. Then a degassed liquid PDMS prepolymer droplet (mixture of the PDMS base and curing agent (Sylgard 184, Dow Corning, USA) with weight ratio of 10:1) is placed onto the slide surface followed by standard spin-coating operation at 3000 rpm for 30 s. After complete curing at 65 °C for 2 h, a CSNPs layer is deposited on the PDMS using a well-studied process called flame synthesis [30], in which the glass slide is mounted right above the burning candle flame core at about 1.5 cm distance and kept for 20 s. Subsequently, another PDMS layer is spin-coated onto the CSNPs layer followed by complete curing. At the same time, another PDMS substrate involving a cylinder cavity is fabricated using a well-known process called soft lithography and a through hole is manually drilled on the cavity sidewall using a commercial hand-held puncher (Harris Uni-core-1 mm, Sigma-Aldrich, Singapore). This PDMS substrate is bonded to the aforementioned PA layer under the assistance of oxygen plasma activation and the fabrication is ended with the peeling of the combined structure from the glass slide.

**Fig. 2:**
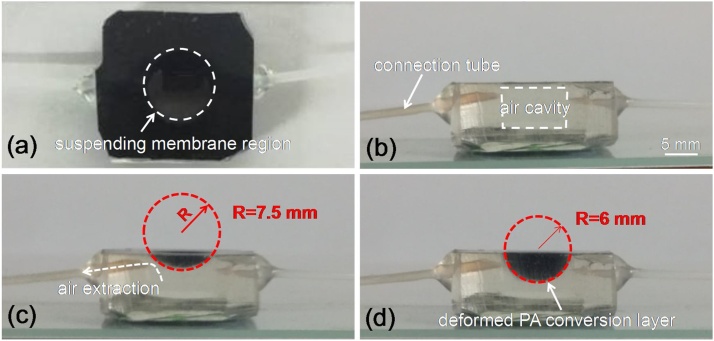
Pictures of the as-fabricated device. (a) top view. (b-d) side view of device under different operation statuses.

Fig. 2 shows the as-fabricated prototype of the proposed focus tunable PA transmitter. The black part in the figure is actually the PA conversion layer due to its high light absorption. It covers a cylinder air cavity (10 mm diameter and 5 mm depth) in a PDMS substrate, forming sealed space for subsequent pneumatic actuation. When extracting air from the cavity via a connection tube, the resultant negative pressure difference *P* with respect to environment will force the center suspending region of the PA conversion layer to deform downward into concave spherical profile dominated by [31]

(1) $$\left\{\begin{array}{l} \frac{d^{2}u}{dr^{2}}+\frac{1}{r}\frac{du}{dr}-\frac{u}{r^{2}}+\frac{1-\nu }{2r}{\left(\frac{dz}{dr}\right)}^{2}+\frac{dz}{dr}\frac{d^{2}z}{dr^{2}}=0\\ \frac{d^{3}z}{dr^{3}}+\frac{1}{r}\frac{d^{2}z}{dr^{2}}-\frac{1}{r^{2}}\frac{dz}{dr}-\frac{12}{d^{2}}\frac{dz}{dr}\left[\frac{du}{dr}+\nu \frac{u}{r}+\frac{1}{2}{\left(\frac{dz}{dr}\right)}^{2}\right]-\frac{P\cdot r}{2D}=0 \end{array}\right)$$

where *u* and *z* represent the radial and axial deflections of the layer, respectively.

**Fig. 3:**
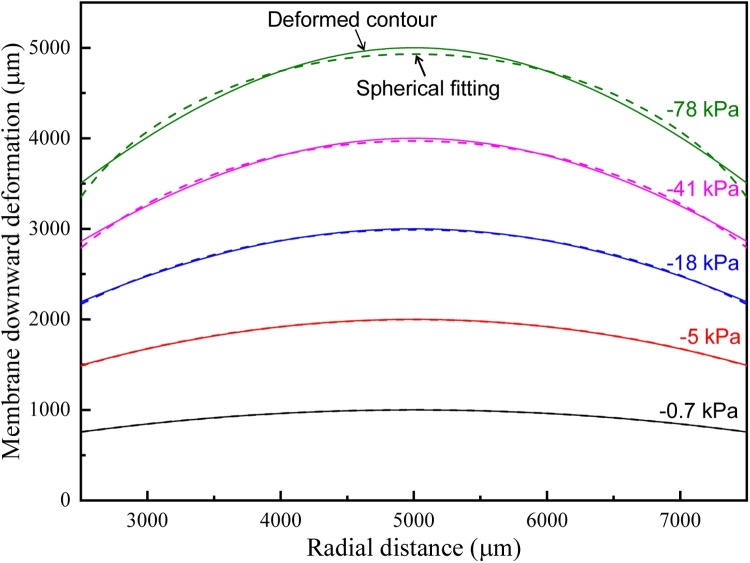
Spherical fitting of the deformed contour of the PA conversion layer under different working conditions.

Through simply controlling the air extraction as well as *P*, the contour of the PA conversion layer with variable radius of curvature can be obtained as clearly seen in figure. Considering the fact that the generated sound field distribution is directly determined by the shape of the PA conversion layer, different focusing capability can be achieved through controlling its deformation. To further study the tuning capability for the focal length, the simulated deformation contours of the suspended PA conversion layer within center region of 5 mm diameter under different working conditions are provided as shown in Fig. 3. From the results of curve fitting, it can be seen that the PA conversion layer will be deformed into spherical like contour in initial status. With the increasing applied pressure, the deformed contour will gradually deviate from spherical shape, therefore deteriorating sound field focusing. Considering the device operation performance, the focal length of sound field can be adjusted from nearly infinity at initial flat status to the shortest value of about 3.3 mm (corresponding to the applied pressure of −40 kPa) given 5 mm operation aperture of PA transmitter.

**Fig. 4:**
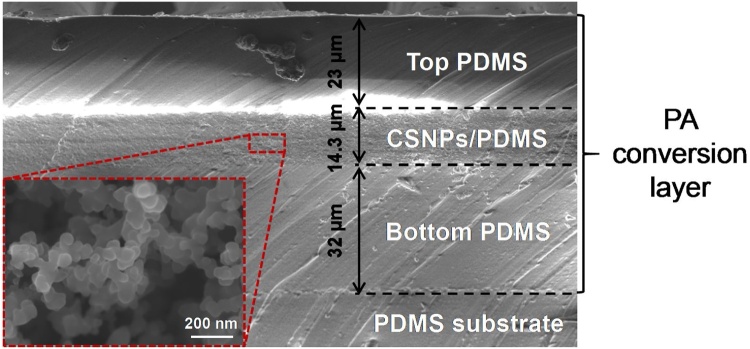
SEM image of the photoacoustic conversion layer.

The SEM images taken from the cross-section of the PA conversion layer are provided in Fig. 4, from which a laminated structure with distinctly visualized interfaces, consisting of a candle soot nanoparticles (CSNPs)/PDMS composite layer sandwiching between two pure PDMS layers, can be revealed. In addition, the as-deposited CSNPs are found to be in a stack structure with size ranging from 30∼60 nm as shown in the inset.

## 3 Experiment results

**Fig. 5:**
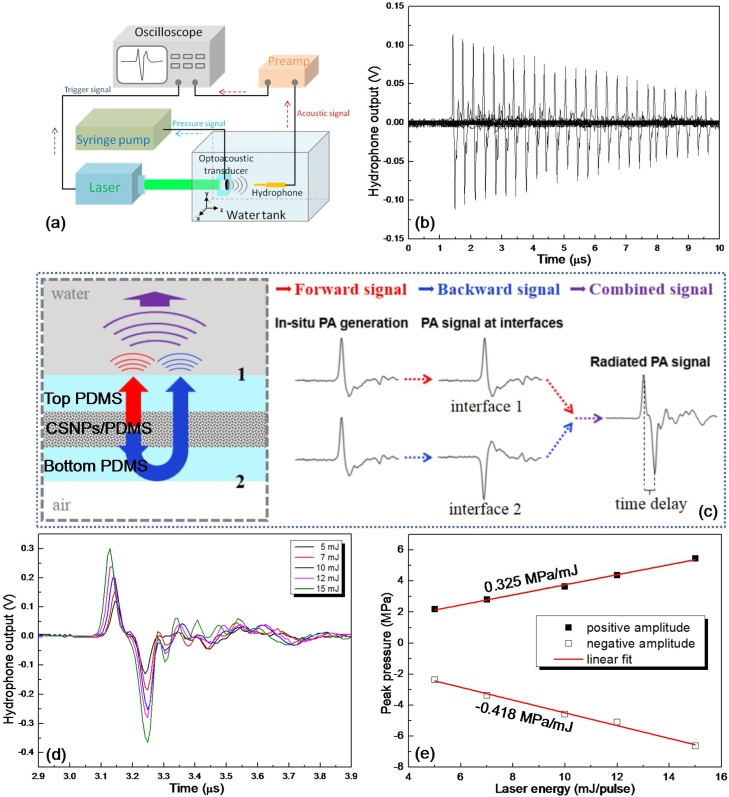
Measured results of the proposed PA transmitter working under initial flat PA conversion layer status. (a) schematic of the measurement setup. (b) the axial transmission of the PA signal.(c) schematic of the PA signal transmission of current PA transmitter. (d) and (e) the change of the PA signal amplitude with respect to the activation laser energy.

In order to characterize the performance of the as-fabricated PA transmitter, experimental setup as shown in Fig. 5(a) is designed. An excitation laser with 532 nm wavelength, 6 ns pulse width and 20 Hz repetition rate (Lapa-80, Beijing Lei Bao Optoelectronics Technology Co., Ltd) is adopted. The device under test is clamped and vertically inserted into a water tank with its rotation axis of symmetry being aligned with the excitation laser beam. Meanwhile, a syringe pumping system is connected to the device using plastic tube for pneumatic actuation. During experiment, once the device surface contour is settled down, the output laser beam with 5 mm diameter is normally incident onto the device through a transparent glass optical window on the water tank sidewall. The radiated PA signal is detected using a piezoelectric needle hydrophone with 0.2 mm diameter (NH0200, Precision Acoustic, UK), which is mounted onto a three-axis translation stage for acoustic field scan (0.5 mm and 0.1 mm sampling intervals along the axial and lateral directions, respectively). The output of the hydrophone is further amplified with a preamplifier and recorded by an oscilloscope (TBS2072, Tektronix, USA). An external trigger signal from the laser source is utilized to synchronize the time series of laser excitation with the oscilloscope data acquisition.

The axial transmission of the PA signal generated by the device operating under initially flat PA layer condition is firstly measured as shown in Fig. 5(b), in which the excitation laser energy is set to be 5 mJ/pulse. It can be seen that the current PA signal demonstrates a symmetric bipolar waveform with nearly the same amplitude in positive and negative phases. In comparison, as for the most common cases where the PA layer is directly deposited onto a rigid substrate, the typical PA signal waveform usually shows a large positive peak followed by a very small negative tail. This significantly improved negative pressure level is mainly induced by the air-backing condition provided by the suspending PA layer design. Fig. 5(c) shows the schematic of the PA signal transmission of current PA transmitter. Upon being illuminated by activation laser, the generated PA signal will in fact propagate in all directions simultaneously, in which the forward and backward transmissions along thickness direction will dominate the outward radiation. In initial status, both of the forward and backward transmission signals demonstrate the same waveform involving a large positive peak followed by a very small negative tail. As for the forward transmission, due to the good acoustic impedance match between the sandwich like PA conversion layer and the surrounding water medium, it can be transmitted through the interface (marked as 1 in figure) between them without experiencing much attenuation whilst keeping the waveform. In contrast, when referring to the backward one, its condition is quite different. Since there exists a large acoustic impedance mismatch between the PA conversion layer and the air, strong reflection will happen when the backward PA signal is transmitted to the air-backing interface (marked as 2 in figure). From the acoustic wave transmission theory, it can be known that current reflection will demonstrate similar amplitude but reversed phase to the incidence due to the trivial acoustic impedance of the air. After that, the reflected signal will be further transmitted through the interface 1 and finally combined with the initial forward transmission signal with a certain time delay determined by transmission distance difference. From the SEM image of the photoacoustic conversion layer as shown in Fig. 4, the thickness of the bottom PDMS is measured to be 32 μm. Considering the round trip transmission in the bottom PDMS layer and its sound speed of 1076 m/s, the time delay is theoretically calculated to be 59.5 ns. In experiment, from the captured PA signal in time domain (see Fig. 5(b)), the time interval between the positive and negative phases is measured to be around 60 ns, agreeing well with theoretical expectation. In addition, from the PA signal captured at 5 mm axial distance away from the initial flat PA layer surface, the conversion efficiency is calculated to be 2.57 × 10^−3^ Pa/(W/m^2^), which is comparable to the mainstream PA material reported [23,32].

Despite of the distinct difference in the signal waveform, monotonous decrease of signal amplitude still can be found along with its axial transmission with decay rate of -3.568 dB/cm due to the divergent acoustic field nature accompany with flat PA layer as well as the transmission loss caused by medium absorption (around -0.031 dB/cm), which is similar to the case of the conventional devices using rigid substrate with flat surface. Moreover, the common sense about the proportional relationship between the PA signal amplitude and the incident activation laser energy in PA area can also be observed. For demonstration purpose, the PA signal captured at the transmission distance of 4.6 mm is selected and the positive and negative acoustic pressure amplitude change rates with respect to the laser energy are measured to be 0.325 MPa/mJ and -0.418 MPa/mJ, respectively, as shown in Fig. 5(d) and (e).

**Fig. 6:**
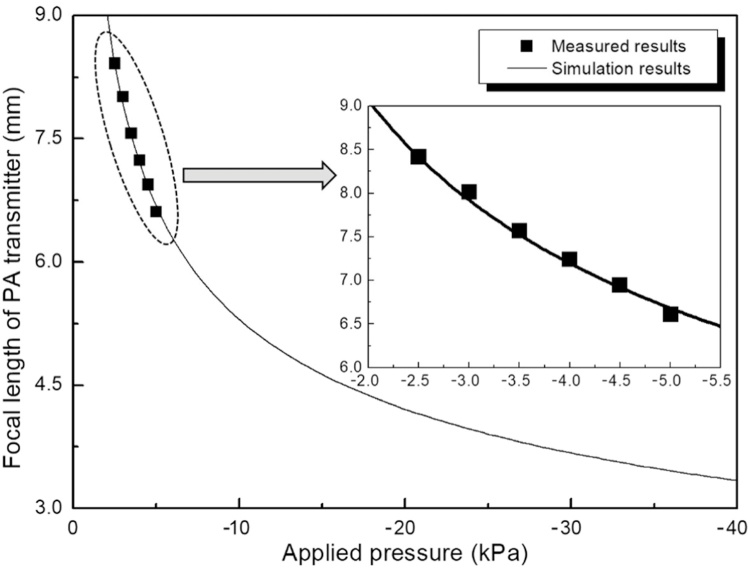
Relationship between the focal length of PA transmitter and applied pressure.

**Fig. 7:**
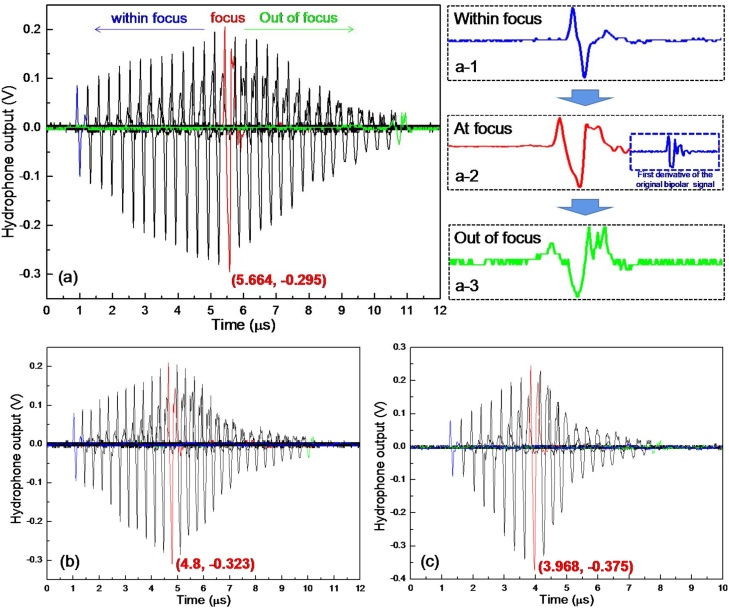
Measured axial transmission of the PA signal under dynamic acoustic focusing operation. (a) the PA transmitter with acoustic focal length of 8.5 mm, the detailed PA signals captured at three different positions along axial direction are also provided: a-1 within focus, a-2 at focus, a-3 out of focus. (b) the PA transmitter with acoustic focal length of 7.2 mm. (c) the PA transmitter with acoustic focal length of 6.0 mm.

Upon dynamic tuning, the air in the device cavity is gradually extracted out using external syringe pumping system and the excitation laser energy is fixed at 5 mJ/pulse, during which the corresponding acoustic field transmissions along the axial direction are recorded as shown in Fig. 6. Although the operation range is relatively small due to the limitation of current experiment condition (limited pressure and scanning length of acoustic field), good agreement between the experimental and simulation results still can be found. For proof of concept demonstration, three different statuses are provided for comparison in Fig. 7. From the results, acoustic field transmission representing distinct focusing characteristic can be observed in all the cases. As for the signal amplitude, progressive increase with initial transmission extension away from the device can be found due to the convergent acoustic field toward focus. After reaching the maximum at the focus, it will be decreased with relatively faster rate along with further transmission instead under the combined effect of the enhanced acoustic field divergence right after focusing and normal transmission attenuation. Although the amplitude change tendency is similar, the details are quite different between these working conditions. The maximum signal amplitude appears at different positions along the time axis. Considering the constant sound transmission velocity of 1500 m/s in water, the corresponding focal lengths are calculated to be 8.5 mm, 7.2 mm and 6.0 mm, experimentally validating the dynamic shift of the sound focus. In addition, larger signal amplitude at the focus as well as its change rate around the focal zone can be obtained when the device works with shorter focal length. This phenomenon agrees well with the acoustic focusing characteristic, in which the dominating factors, namely the focal gain and the divergent angle of the acoustic field, for PA signal amplitude and its change rate, respectively, both are highly correlated to the f-number of the device, which in turn is determined by the focal length given the same device aperture size.

As for the focused acoustic transmitter, the focal gain *G*, defined as the ratio of the signal amplitude at the focus to that on the transmitter surface, is one of the most important parameters for performance evaluation and it can be described by [33]

(2) $$G=\frac{2\pi }{\lambda }f(1-\sqrt{1-\frac{1}{4f_{N}^{2}}})$$

where *λ* is the wavelength of the acoustic signal, *f* is the focal length, and *f_N_=f/D* is the f-number of the device, *D* is the device aperture.

**Fig. 8:**
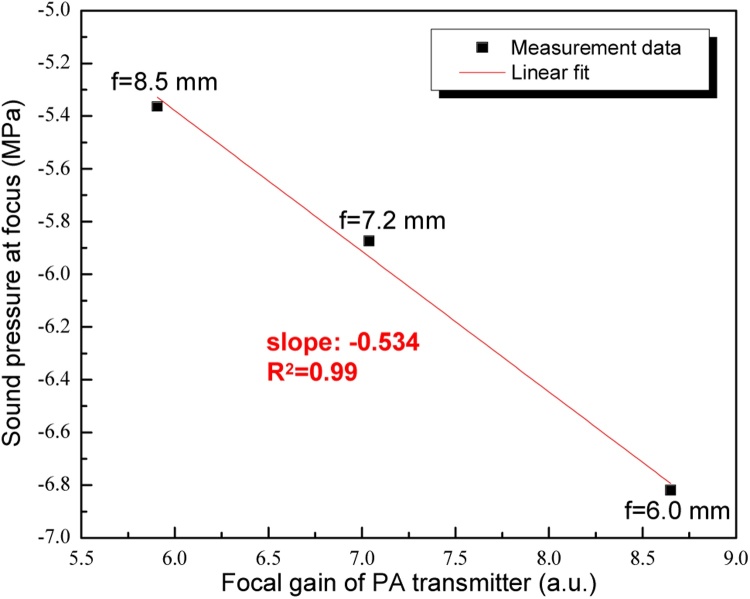
Change of the sound pressure at the focus as a function of the focal gain of the PA transmitter.

From which, the focal gains of the device operating under three different conditions as shown in paper can be calculated to be 5.91, 7.04 and 8.65 when the focal lengths are 8.5 mm, 7.2 mm and 6.0 mm, respectively. After extracting the negative pressure amplitudes at individual foci (the pressure amplitude can be calculated from the hydrophone output using its detection sensitivity of 55 mV/MPa), good linear relationship between the amplitude and the focal gain can be clearly observed (see Fig. 8), agreeing well with theoretical expectation. As for the focused PA transmitter, the acoustic signal amplitude at the focus can be treated as the signal amplitude on the transmitter surface multiplied by its focal gain. From this consideration, the slope in the figure should represent the negative acoustic pressure generated at current PA transmitter surface. Compared with that of the initial flat status, this value is a bit smaller. It is because that upon deformation, the stack of the CSNPs in the PA conversion layer will become loose, thus lowering its light absorption as well as the resultant PA signal amplitude.

**Fig. 9:**
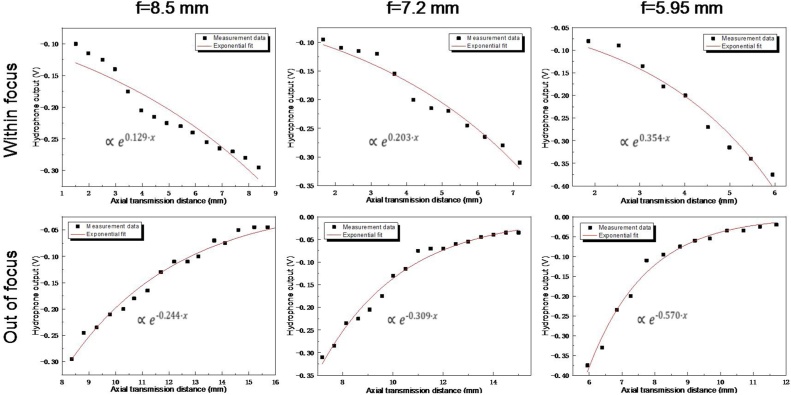
Change of negative sound pressure along with axial transmission.

From the axial transmission results shown above, the full width at half maximum (FWHM) of corresponding acoustic pressure distribution profiles are calculated to be 8.23 mm (f = 8 mm), 6.13 mm (f = 7.2 mm) and 3.75 mm (f = 6.0 mm), respectively. At the same time, nearly exponential change of the PA signal amplitude (both positive and negative phases) can also be found, in which the signal amplitude will be firstly increased exponentially with transmission toward the focus (called within focus region). After passing the focus, exponential decrease with larger change rate will be happened with further transmission (called out of focus region). At the same time, as for the same device operating under different focusing statuses, the change tendencies are also quite different. For better disclosing the focusing dependent amplitude change, the negative signal amplitudes at each measurement points along with the axial transmission are specially extracted out and exponential fit is subsequently performed as shown in Fig. 9. It can be seen that with the acoustic focal length of the PA transmitter being adjusted to shorter end, larger change rate of the PA signal amplitude along with axial transmission can be obtained in both regions. Considering the fixed operation aperture diameter of 5 mm, with the tuning of the focal length, the convergent (within focus) or divergent (out of focus) angle θ of the acoustic field will also be changed

(3) $$\theta =2\cdot \arcsin\left(2.5/f\right)$$

Obviously, with the decrease of the working focal length, the resultant acoustic field will become more convergent/divergent. As a result, larger change of its distribution area will be induced. Since the total acoustic energy is constant, the amplitude will be directly correlated to its distribution area.

It should also be noted that different from above mentioned device operation under flat PA layer, the symmetric bipolar PA signal waveform in initial status will gradually transform into a quasi-monopolar one toward sound transmission to the focus. This waveform transformation is mainly caused by the fact that the temporal profile of the sound pressure pulse at the focus can be approximated by the first derivative of the signal profile close to the device surface (This can be validated from the first derivative result of the bipolar PA signal captured near device surface as shown in Fig. 7 (a-2) inset). Owing to this transformation, significantly enhanced negative phase amplitude can be successfully achieved as well. After passing through the focus, this quasi-monopolar waveform will be preserved except for the decreasing amplitude along with continuous transmission.

**Fig. 10:**
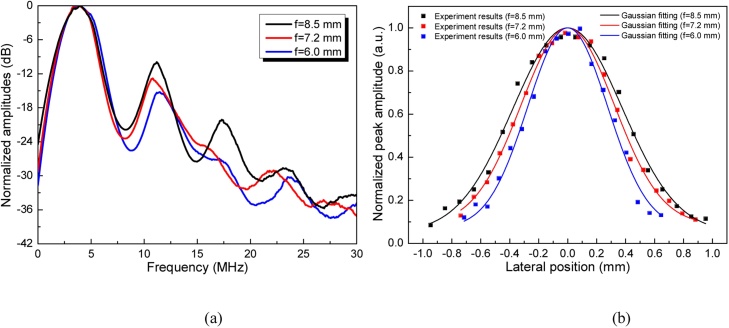
Measured PA signal during dynamic tuning. (a) Frequency responses of the generated PA signal at different foci. (b)Lateral acoustic field profiles in the focal plane.

In order to further reveal the PA signal feature, FFT is performed on the time-domain signals captured at different foci and the resultant frequency responses are provided in Fig. 10(a), from which almost the same central frequency of 3.75 MHz and 88 % bandwidth at −6 dB can be obtained in all the cases, demonstrating good performance consistency during dynamic tuning. Besides the axial transmission scan, the lateral acoustic field profile in the focal plane is also studied as shown in Fig. 10(b). It’s obvious that all of the acoustic fields demonstrate Gaussian-like amplitude distribution as theoretical expectation. Meanwhile, the FWHM of individual profiles are calculated to be 920 μm (f = 8.5 mm), 810 μm (f = 7.2 mm) and 685 μm (f = 6.0 mm), respectively. This phenomenon - the shorter the focal length, the smaller the focal spot - is consistent with the classic acoustic focusing theory and mainly attributed to the f-number determined acoustic field diffraction.

**Fig. 11:**
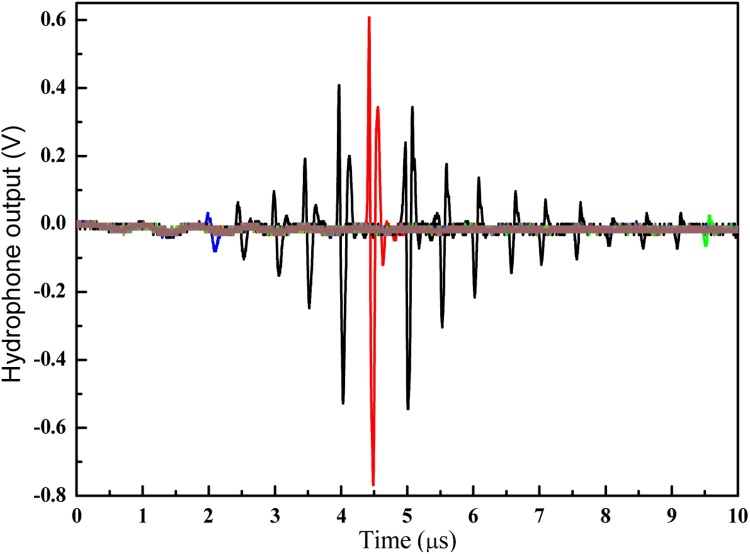
Measured axial transmission of the PA signal from the focused PA transmitter operated under 10 mm aperture size.

Moreover, the effect of the transmitter aperture size on its performance is also studied, in which full aperture size, namely 10 mm, is used instead. In this case, considering the enlarged illumination area, the excitation laser energy is increased to 10 mJ/pulse and the axial scanning result of the acoustic field generated from the device under focusing status is provided in Fig. 11. Compared with aforementioned case using 5 mm aperture, besides the higher acoustic pressure amplitude, faster change rate of acoustic pressure around the focus can also be found. This more distinct focusing characteristic is mainly caused by the increased focal gain associated with larger operation aperture size (with the increasing aperture size from 5 mm to 10 mm, the focal gain will be increased from 8.65 to 42).

**Fig. 12:**
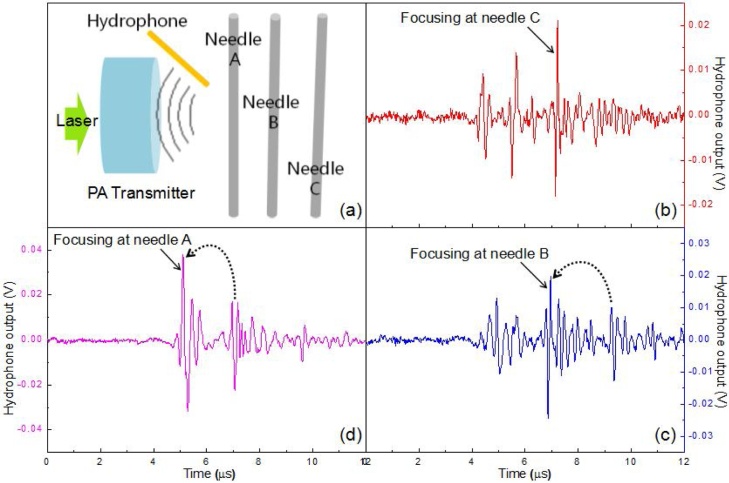
Pulse-echo experiment. (a) schematic of the experiment setup. (b-d) captured echo signals in time domain during dynamic focusing experiment, in which the acoustic field is made focused onto each needle from far to near.

To qualitatively demonstrate the application advantage imposed by the dynamic acoustic focusing capability, a unique pulse-echo experiment is designed. As shown in Fig. 12(a), three steel needles with 0.6 mm diameter are arranged along the transmission axis of the proposed device with nearly 1 mm interval. In experiment, the laser energy is set to 10 mJ/pulse and the device is dynamically actuated to make the acoustic field focusing onto each needle from far to near step by step, during which a hydrophone is employed to detect the echo signals reflected by the needles. From the captured real time signals provided in Fig. 12(b)-(d), it is clearly seen that there are three obvious echo signals aparting certain time interval in all the cases, representing the reflections from the targeted needles. Considering the proportional relationship between the transmission length and the transmission time, one-to-one correspondence can be easily built between signal and target. In the beginning, the acoustic focus is made falling onto the needle C. Due to the concentrated acoustic energy onto the needle surface, the largest amplitude can be obtained on its reflection signal irrespective of its longest transmission time as well as transmission distance. With the dynamic shift of the acoustic focus onto different needles, the corresponding echo signal will express distinctly larger amplitude compared to others. This significantly strengthen signal amplitude associated with acoustic focusing helps to improve the signal to noise ratio at the focus region, which will benefit the applications especially for the cases of large penetration depth and low contrast imaging. Undoubtedly, the dynamic tuning capability on acoustic focusing will further extend the effective operation range and enhance applicability.

Considering the device structure, its tuning speed is actually determined by the frequency response characteristic of the suspended composite membrane. As for current device design, the tuning speed is around 20 ms, which is comparable to motorized translation stage but the system complexity as well as weight and volume will be distinctly reduced especially for large tuning range cases.

## 4 Conclusions

In summary, the first real-time dynamic acoustic focusing realization in the PA transmitter is experimentally demonstrated using a novel device design concept. Different from conventional PA transmitters, a sandwich-like suspending PA conversion layer covering a cavity to build an integrated pneumatic actuator is designed. Taking advantage of the specific mechanical property and the good flexibility of the PA layer given by the suspension treatment, it can be deformed into concave spherical profile with relatively large tuning range upon pneumatic actuation. Considering the PA conversion layer shape determined sound field distribution, dynamic acoustic focusing along axial direction has been successfully achieved with simple hardware configuration and easy control, providing a feasible and prospective solution to address the technical challenge faced by the PA society. Moreover, owing to the air-backing condition associated with the suspending PA structure, acoustic signal with significantly improved negative pressure level can also be obtained especially at the focus, bringing additional advantage for ultrasound cavitation-based applications. Besides the currently adopted CSNPs/PDMS composite, other PA conversion materials can also be easily involved into the proposed structure through properly modifying the fabrication process, thus demonstrating excellent universality and adaptability.

## Funding

This work was supported by the National Natural Science Foundation of China (NSFC) (61875244, 11774117); Ministry of Education of the People’s Republic of China (MOE) (2016JCTD112).

## Declaration of Competing Interest

The authors report no declarations of interest.
